# Spider-Inspired HCCapture: Beware That What You Are Writing on Mobile Devices Is Becoming Prey for Spiders

**DOI:** 10.3389/fbioe.2022.858961

**Published:** 2022-04-19

**Authors:** Wei Fu, Tingting Zhu, Jing Chen, Peidong Jiang, Kun He, Cheng Zeng, Ruiying Du

**Affiliations:** ^1^ Department of Information Security, Naval University of Engineering, Wuhan, China; ^2^ School of Cyber Science and Engineering, Wuhan University, Wuhan, China; ^3^ School of Marxism, Wuhan University of Science and Technology, Wuhan, China

**Keywords:** mobile device, motion sensor, information leakage, handwriting speculation, neural network

## Abstract

On mobile devices, the most important input interface is touchscreen, which can transmit a large amount of sensitive information. Many researchers have proven that sensors can be used as side channels to leak touchscreen interactive information. The research of information leakage in the restricted area has been relatively mature, but in the unrestricted area, still there are two issues to be solved urgently: chirography difference and posture variation. We learn from the way spiders perceive prey through the subtle vibrations of their webs; an unrestricted-area handwriting information speculation framework, called spider-inspired handwriting character capture (spider-inspired HCCapture), is designed. Spider-inspired HCCapture exploits the motion sensor as the side-channel and uses the neural network algorithm to train the recognition model. To alleviate the impact of different handwriting habits, we utilize the generality patterns of characters rather than the patterns of raw sensor signals. Furthermore, each character is disassembled into basic strokes, which are used as recognition features. We also proposed a user-independent posture-aware approach to detect the user’s handwriting posture to select a suitable one from some pretrained models for speculation. In addition, the Markov model is introduced into spider-inspired HCCapture, which is used as an enhancement feature when there is a correlation between adjacent characters. In conclusion, spider-inspired HCCapture completes the handwritten character speculation attack without obtaining the victim’s information in advance. The experimental results show that the accuracy of spider-inspired HCCapture reaches 96.1%.

## 1 Introduction

As the most popular human–computer interaction interface on mobile devices, touchscreen transmits a plenty of sensitive information from users to mobile applications. For example, users can enter passwords by tapping virtual keyboards, unlock devices by drawing patterns, and input messages by handwriting.

At present, numerous research works have confirmed that touchscreen information (e.g., PIN and unlock pattern) can be speculated by some malicious background applications. Roughly speaking, even though those background applications cannot directly obtain the touchscreen information, they can access the signals of some built-in sensors, such as accelerometer and gyroscope. By exploiting those sensors as a side-channel, touchscreen information can be speculated, such as passwords tapped by users on the virtual keyboard (Xu et al., 2012; Das et al., 2018; Zhao et al., 2019). By far, most research work only focus on the *restricted-area* input interface, in which touchscreen information is entered by tapping a specified position/area on the touchscreen, such as the virtual keyboard (Spreitzer et al., 2018a; Mehrnezhad et al., 2018) and the pattern lock screen (Aviv et al., 2012; Zhou et al., 2018).

On the other hand, more and more touchscreens support *unrestricted-area* input interface, in which users can tap any position/area on the touchscreen for input, such as gesture control and handwritten input. Mehrnezhad et al. (2016) have shown that primitive operation actions (e.g., click and scroll in gesture control) on touchscreens can be recognized by analyzing sensor signals. Nevertheless, the speculation for more complex and meaningful inputs, such as handwritten contents, has not been sufficiently discussed. One issue is that victims are usually *unknown* to attackers before attacks, but the speculation model is built on the training data from the *known* users. Meanwhile, we observe that each person has his/her own handwriting habits, such as writing strength, sequence, and speed, called *chirography difference*. The other is that the sensors’ signals and noise patterns vary under different holding postures (e.g., sitting and standing), since the victim’s limb jitter and handwriting strength change significantly under different postures, called *posture variation*. Moreover, posture variation also needs to be handled when the victim’s data are not included in the training process of the speculation model.

In this study, we learn from the way spiders perceive prey through the subtle vibrations of their webs, design a framework that runs on Android called spider-inspired handwriting character capture (spider-inspired HCCapture) to speculate handwriting contents in the unrestricted area. The main idea of spider-inspired HCCapture is to track the changes of sensor signals caused by handwriting actions on the touchscreen, and recognize the patterns for each character through the model trained by the neural network algorithm. To solve the chirography difference issue, spider-inspired HCCapture utilizes a generality pattern of characters (i.e., stroke number), which enables character speculation without collecting the target victim’s training data in advance. Regarding the posture variation issue, we propose a user-independent posture-aware approach based on the correlation comparison of holding posture features, to dynamically perceive the victim’s posture. With the perceived posture, spider-inspired HCCapture can adjust the speculation model to improve the speculation accuracy.

The contributions of this article are summarized as follows:

•   We propose to utilize generality patterns of characters (number of strokes and type of strokes) to alleviate the dependency on collecting training data from victims in advance and reduce the impact of chirography difference on speculation accuracy. Specifically, with the generality patterns, spider-inspired HCCapture can divide the character set into different character clusters and dramatically narrow the scope of speculation.
•   We build diverse speculation models in spider-inspired HCCapture according to different holding postures detected by a user-independent posture-aware approach. Each speculation model is trained with the sample sensor signal data gathered under a corresponding holding posture. Therefore, spider-inspired HCCapture is able to complete handwritten information speculation with competitive accuracy in more practical scenarios. Experiment results also show that the speculation accuracy of spider-inspired HCCapture is better than that of the universal speculation model.
•   We implement a spider-inspired HCCapture prototype using off-the-shelf mobile devices with the Android platform. In the 3-fold cross-validation experiment, the accuracy of spider-inspired HCCapture speculated characters reached 96.1%.

The rest of the article is structured as follows. First, Section 2 summarizes related work. Second, we introduce the preliminaries in Section 3. Section 4 details the design of spider-inspired HCCapture. Then, the performance of spider-inspired HCCapture in different conditions is shown in Section 5. At last, Section 6 concludes our study.

Spider-inspired HCCapture is an improvement of J. Chen et al.’s work (Chen et al., 2021a). Compared to the previous version, we have made three major improvements. First, under the premise of noting the difference in the number of strokes of characters, we divide the strokes into eight basic types and label them. In spider-inspired HCCapture, we use the detected strokes to reconstruct the characters, instead of directly using the classifier to predict the entire signal of a character. Second, we added lowercase characters, which makes spider-inspired HCCapture more practical. Thanks to a more fine-grained model, the accuracy of the experiment is further improved, despite the doubled character set. At the same time, considering that there may be relevance between handwritten adjacent characters, we also introduced a Markov model. At last, corrections were made to the places where the previous experiment was not rigorous. We strictly exclude the tested data from the training set, and use a 3-fold crossover experiment for verification.

## 2 Related Work

Mobile devices’ restricted-area information could be classified as two categorizations: virtual keyboard input and pattern lock (Spreitzer et al., 2018a). Cai et al. (Cai and Chen, 2011) first proposed the possibility of eavesdropping virtual keyboard input *via* embedded sensors in a smartphone. They developed TouchLogger, which can monitor the orientation signals and extract features from these signals to infer key-press information. TouchLogger’s accuracy can reach 70% when the recognized characters are only numbers. Similar to this work, Xu et al. (Xu et al., 2012) recorded gyroscope signals to infer user input and PIN code. Ping et al. (Ping et al., 2015) proposed a method to infer even longer input.

Mehrnezhad et al. (Mehrnezhad et al., 2018) presented a threat of eavesdropping users’ PINs by recording the sensors’ signals from a web page. They proposed PINlogger.js which is a JavaScript-based side-channel attack embedded in a web page, recording the sensor signal changes while a user inputs the sensitive information on other web pages. However, as the mobile operating system gradually restricts collecting sensor signals from web pages, this attack method is not effective any longer. All of these research works only focus on the disclosure of restricted-area information, and only a few number of researchers study the threat to unrestricted-area information based on sensor side channel.

Currently, only a few studies have focused on the leakage of unrestricted-area information. Researchers have shown that simple touch actions including clicking, scrolling, zooming, and holding can be recognized *via* analyzing motion and orientation sensors’ signals (Mehrnezhad et al., 2016; Spreitzer et al., 2018b). However, handwritten information is generally more complicated to be restored than simple touch actions (Yu et al., 2016; Du et al., 2018). Yu et al. (2016) showed that victims’ personal information could be reinstituted by the audio signals of mobile devices. Their system, named WritingHacker, recognized characters with an off-line trained model according to different strokes of characters. Their experiments revealed that the accuracy of word recognition was around 50–60*%* under certain conditions.

Mobile security involves many fields. An endless stream of attacks makes researchers pay attention to the research of vulnerabilities in electronic products and smart phones (Niu et al., 2008; Zhou et al., 2016; Hur and Shamsi, 2017; Delgado-Santos et al., 2021). In order to get the optimal solution, bionic algorithms derived from nature often provide a novel research idea (Liu et al., 2021a; Xu et al., 2022; Zhang et al., 2022). In recent years, deep learning models have provided new solutions to traditional problems (Jiang et al., 2021a; Jiang et al., 2021b; Tao et al., 2022; Yun et al., 2022). Similarly, mobile security is closely related to society, and the game theory in sociology also has a certain reference value (Chen et al., 2021b; Chen et al., 2021c; Chen et al., 2021d). In addition to the touchscreen, the methods used in manipulator recognition in mechanical and industrial fields (Sun et al., 2020; Liu et al., 2021b; Hao et al., 2021; Xiao et al., 2021; Yang et al., 2021) are also relevant to this study.

## 3 Preliminaries

This section introduces the necessary preliminary research and threat models.

### 3.1 Targeted Vulnerable Apps

Although the prototype of spider-inspired HCCapture in this article is implemented on the Android platform, our spider-inspired HCCapture framework can also be used on other mobile platforms, such as iOS. A recent analysis shows that the total number of apps is 2.8 million in Google Play and 2.2 million in Apple App Store in 2019 (Buildfire, 2019). Since spider-inspired HCCapture is a system to speculate handwritten information, the targeted vulnerable apps are mainly handwriting-related apps. To understand the distribution of handwriting-related apps on the markets, we have conducted a ubiquitous survey on mobile application markets (including Android and iOS platforms) using the crawler technology and the third-party data sets. The results are shown in Table 1.

**TABLE 1:** Survey of handwriting-related apps.

Markets	Downloads/apps	Example app
Google Play	347,014,420	Google handwriting input
AnZhi	4,545,245	Sogou input method
AppChina	455,663	Chinese handwriting recog
WanDouJia	224,254	ABC handwriting
Apple Store	1,381	NoteBook+

We have investigated four application markets for the Android platform and designed a set of crawlers based on scrapy (Scrapy, 2022) to analyze downloads of handwriting-related apps. Each crawler has different rules for different markets’ websites. We also perform a lexical analysis of apps’ description in these markets and select the apps whose description contains the keywords like “handwriting” or “scrawl” as our statistical objects. Since the Apple App Store does not have download statistics, we count the number of handwriting-related apps in the App Store based on a third-party data set (Qimai, 2019). Table 1 shows that the total downloads of targeted vulnerable apps in Google Play is over 300 million and the number of apps in the Apple Store (including the United States and China) is up to 1,381, which draws a conclusion that there are enormous targeted vulnerable apps which could potentially be compromised by spider-inspired HCCapture.

### 3.2 Motion Sensor Selection

Modern mobile devices have been equipped with many types of sensors to perceive the physical world. Each type of sensors only focuses on measuring its interested properties. For instance, the magnetic sensor measures the magnetic field signals around the mobile device, and the light sensor measures the light intensity. With the continuous development of sensor hardware, equipment manufacturers are also equipped with more and more sensors on their mobile phones to enhance their intelligence. For example, the distance sensor is usually used to determine whether the user is holding the phone close to their head *via* infrared rays.

**FIGURE 1:**
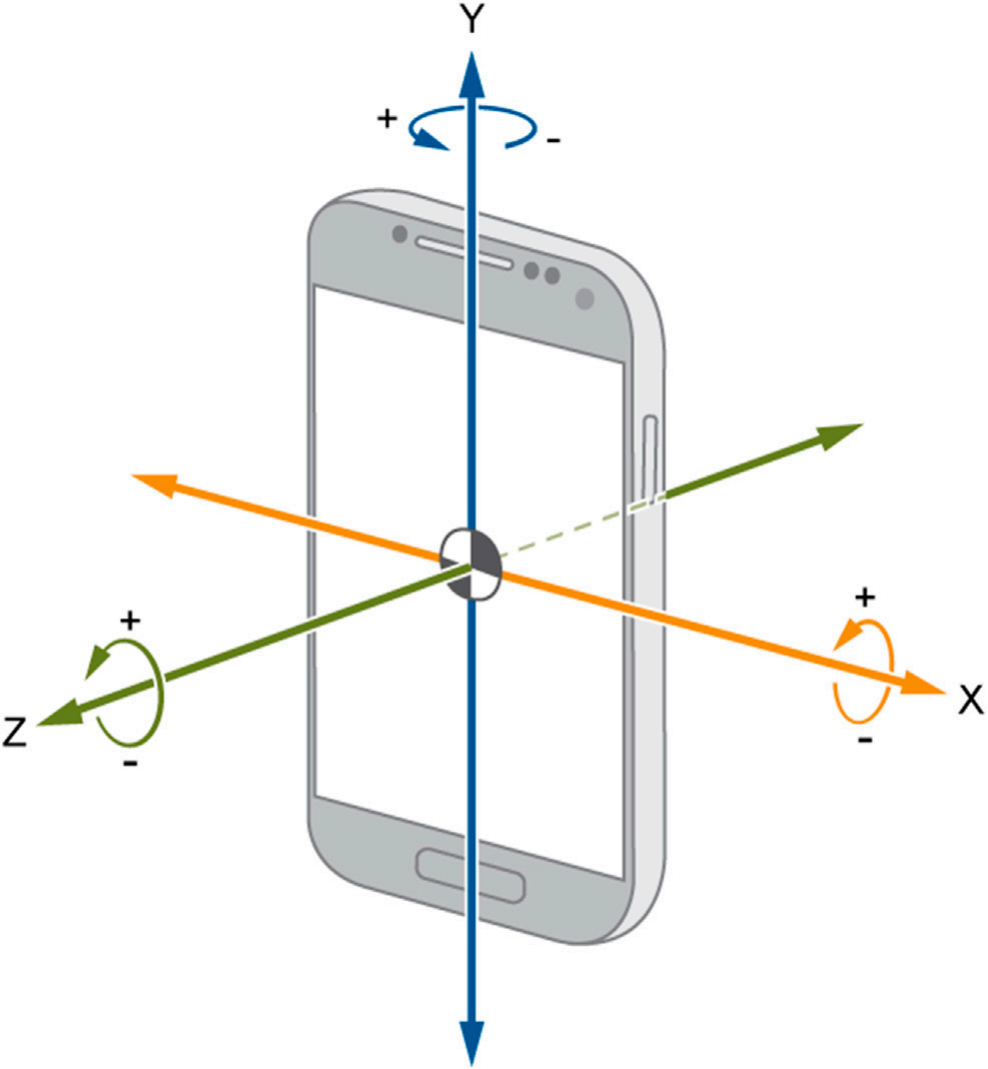
Accelerometer and gyroscope.

However, for the existing mainstream mobile phone operating systems, such as Android and iOS, access to general sensor data does not need application for permission, so we can freely obtain motion sensor data. In spider-inspired HCCapture, in order to speculate the handwritten information, we need to precisely perceive the mobile devices’ movement caused by handwriting actions. Specifically, we employ two commonly used motion sensors, accelerometer and gyroscope, to measure the acceleration and angular velocity of a device separately. Conceptually, these sensors provide signals at a given frequency which contain the data in three dimensions (denoted as X, Y, and Z, respectively, in Figure 1) and its corresponding timestamps.

### 3.3 Threat Model

We assume that a malicious app is developed and gets listed in the application market, which may pretend as a mobile game app. Once installed on a user’s mobile device, this malicious app will record motion sensors’ signals. To get unnoticed by the user, the malicious app tries to be as stealthy as possible by running in the background, and only records sensors’ signals when the user runs a handwriting-related app in the foreground. Existing work has proposed a way to determine the running app *via* power analysis (Chen et al., 2017a; Chen et al., 2017b; Qin and Yue, 2018). Thus, combining our work in Section 3.1, we can start monitoring sensors’ signals only after determining that the foreground app is what we are interested in.

The malicious app requires network permission, which is usually allowed by default on the *Vanilla Android*, a system not deeply customized. And this permission is so common in today’s game apps that it usually will not gain users’ attention. At the same time, all collected data are stored in *App-specific storage* to reduce unnecessary storage permission.

Finally, the malicious app is assumed to be running on the Android system with tolerance in the background policy. When the user actively removes the app from the recent task, it can simply reside in the background through the service. Spider-inspired HCCapture does not compete with the deeply customized Android, especially in China, for background survival.

## 4 Implements

After weaving a cobweb, the spider will wait on or near the cobweb. Once the prey is stuck by the spider silk, the collision or vibration of the prey will be transmitted to the cilia on the spider’s feet through the cobweb. The spider will judge the position of the prey by feeling the vibration of the spider silk from different directions. Inspired by the mechanism of spider predation, an approach on detecting handwritten characters on mobile devices by using motion sensors is designed.

**FIGURE 2:**
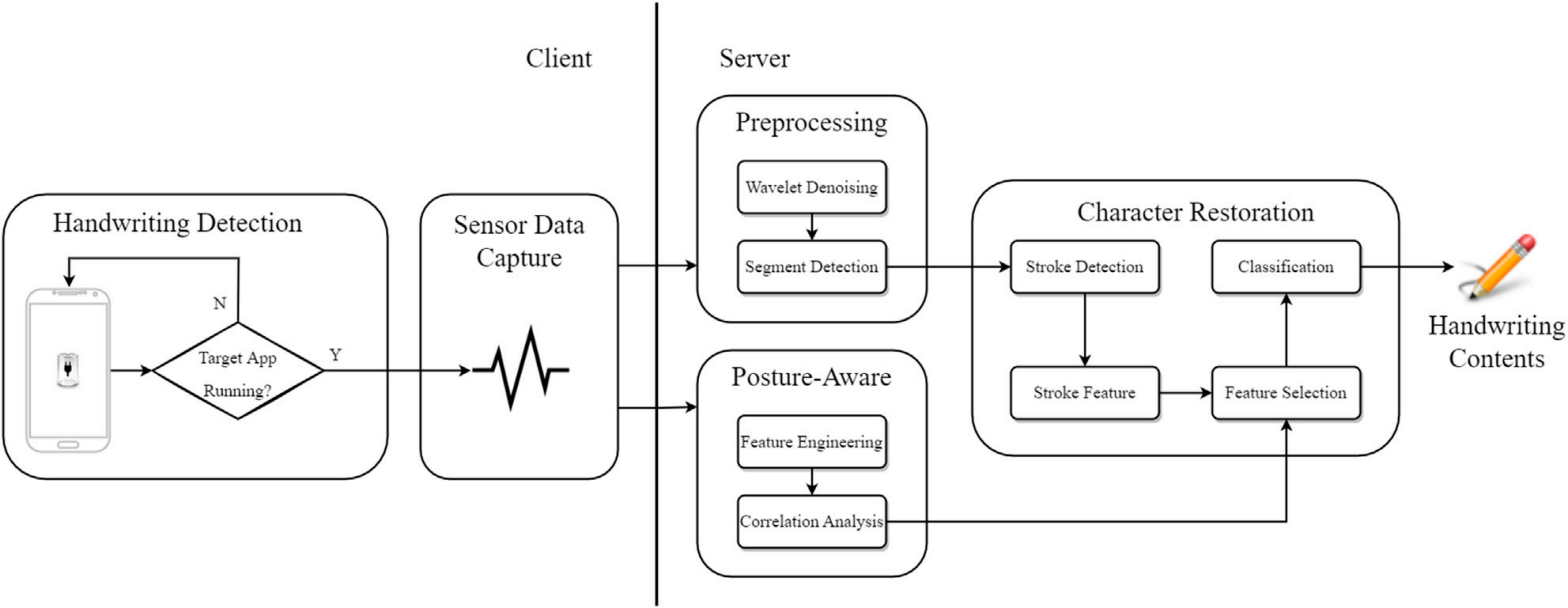
System architecture of spider-inspired HCCapture.

This section describes the specific implementation of spider-inspired HCCapture, and its overall structure is shown in Figure 2.

### 4.1 Handwriting Detection

Handwriting detection aims to detect when a victim is handwriting information in an application so that spider-inspired HCCapture can immediately begin recording motion sensors’ signals to capture the fingertip motions on the touchscreen.

Detecting handwriting actions is very different from detecting unlocking screen actions. Unlocking screen actions can be perceived by receiving the corresponding system broadcast (Zhou et al., 2018), while handwriting actions do not have this observable system broadcast. Handwriting actions usually occur in input methods or some note-taking applications, but the system does not broadcast any information when victims use these applications. Meanwhile, other applications cannot use conventional approaches to identify what a foreground application is.

Fortunately, different applications use different components of a device (e.g., touchscreen, CPU, Wi-Fi, and Bluetooth) and have different usage patterns, which result in distinguishable power consumption profiles. We adopted the solution of Y. Chen et al. (Chen et al., 2017b). By analyzing the power consumption on mobile devices, we can detect when input methods or some note-taking applications are active, and begin capturing the corresponding handwriting actions. Since we do not need monitoring motion sensors’ signals all the time, spider-inspired HCCapture can not only reduce the power consumption but also avoid collecting irrelevant signals.

### 4.2 Sensor Data Capture

Once a handwriting action is detected, spider-inspired HCCapture leverages the sensor signal monitoring interface provided by the Android system, to conduct real-time monitoring of motion sensors’ signals during the handwriting process. Spider-inspired HCCapture collects the signals from two sensors: *accelerometer*, which can measure device vibration and acceleration changes caused by handwriting actions, and *gyroscope*, which can estimate the rotation and deflection of the device caused by handwriting actions. Finally, spider-inspired HCCapture uploads the collected sensor signals data to a remote server stealthily for analysis.

### 4.3 Preprocessing

The main tasks completed at this stage are denoising, and identifying and extracting the signal stream corresponding to each character.

1) Wavelet denoising: The collected sensors’ signals usually contain a mass of background noise which may greatly affect the subsequent analysis of handwriting actions. To filter the noise from these raw sensors’ signals and restore the real signal fluctuations caused by handwriting actions, we utilize the wavelet denoising for filtering.

**FIGURE 3:**
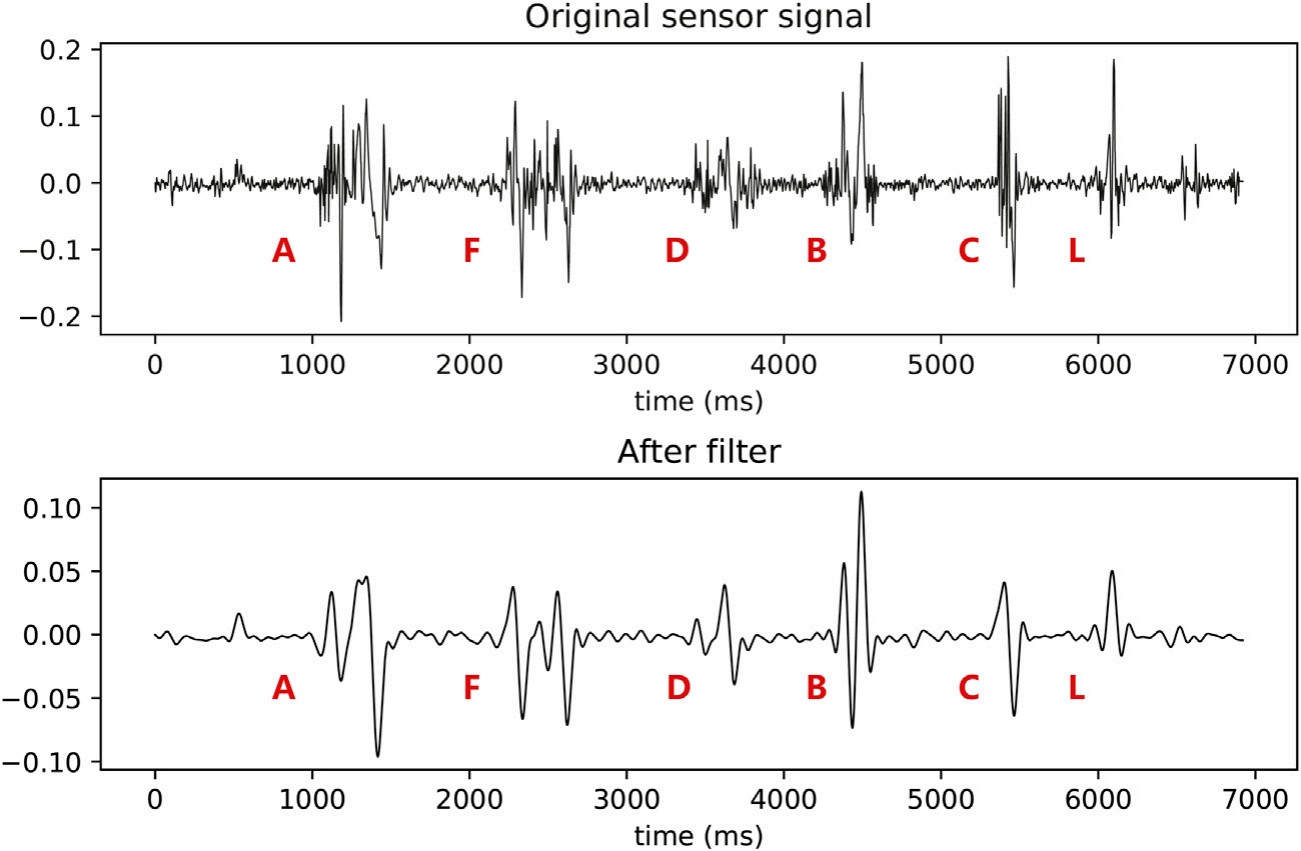
Original vs. wavelet denoised sensor signals of handwriting action.

Figure 3 shows the gyroscope’s signals for handwriting actions of character “A,” “F,” “D,” “B,” “C,” and “L.” After wavelet denoising, we can find the actual change pattern of signal fluctuations during the handwriting process for different characters. More specifically, the difference in the number of peaks in Figure 3 can be observed, as the characters have different strokes. For example, the signal of character “A” has three strokes, and character “C” has only one stroke. The number of strokes observed here is an important basis for us to solve the *chirography difference* issue (see Section 4.5 for details).

2) Segment detection: The goal of segment detection is to extract individual signal segments generated by the handwriting action of a single character from the above denoised signal stream. Generally, it is difficult for victims to keep their arms and palms still for a long time, and therefore, the collected sensors’ signals would be unstable. Extracting the target signal segment from the unstable signals is the first step to implement the handwritten character speculation.

One trivial method is to set a constant threshold. When the amplitude of signals exceeds this threshold, these signals are regarded as in the same signal segment. However, it is challenging to set such an immutable threshold in advance, since the amplitude of noise signals changes constantly in practice. Moreover, since everyone’s handwriting habits are different, the resulting signal fluctuations are distinct.

In the previous work by Shen et al. (2015), some signal segment detection algorithms have been proposed for eavesdropping *restricted-area* information. The main idea of these algorithms is to detect the remarkable fixed patterns in sensor signal changes of the *restricted area*. For instance, in virtual keyboard input, all character information is typed in application by tapping actions, and the signal fluctuations are extremely similar. Nevertheless, entering characters using handwriting needs users to slide significantly distinct trajectories on the touchscreen. There is also a handwriting signal segment detection algorithm in the study by Yu et al. (2016). It is used to extract the handwriting signal segments from the continuous sound streams in a quiet environment, and the experimental facilities remain stationary in their experiment. Unfortunately, the motion sensors’ signals collected in our experiment may have more occasional noise caused by the unintentional shake of victim’s arms and palms.

In this article, we quoted the modified constant false alarm rate (mCFAR) algorithm proposed by J. Chen et al. (Chen et al., 2021a), to identify handwriting actions from the motion sensors’ signals. The mathematical description of our algorithm is shown as Algorithm 1.

Algorithm 1 mCFAR

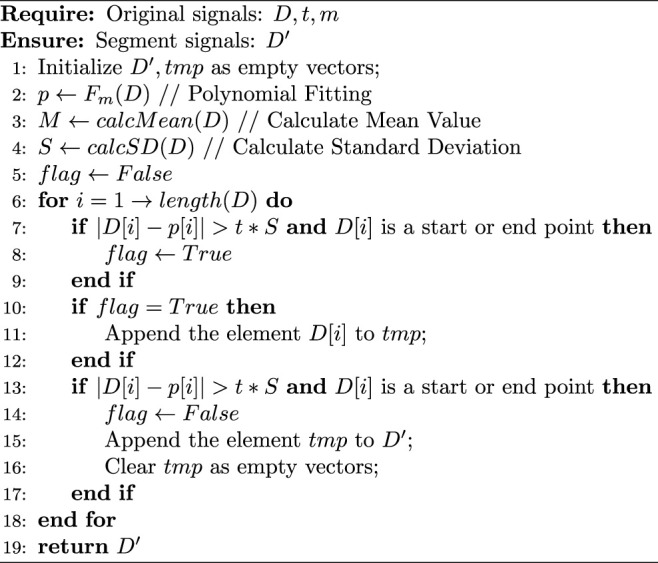

### 4.4 Posture-Aware Analysis

In this phase, spider-inspired HCCapture perceives the victim’s holding posture by analyzing the background noise in the collected sensors’ signals.

In recent years, sophisticated sensors such as accelerometer and gyroscope equipped in mobile devices have provided the opportunity for continuous collecting and monitoring signals for human activity recognition (HAR) (Chen et al., 2017c; Montero Quispe et al., 2018; Wang et al., 2019). After extracting various features from raw sensors’ signals, supervised machine learning algorithms are used to train and validate the results. This is the basic flow of the traditional human activity recognition research.

However, the features used in those studies are not user-independent, resulting in insufficient generalization of the trained model. How to use the model trained by the known users’ posture data to identify the unknown users’ posture data is the challenge (i.e., posture variation) that needs to be solved.

**FIGURE 4:**
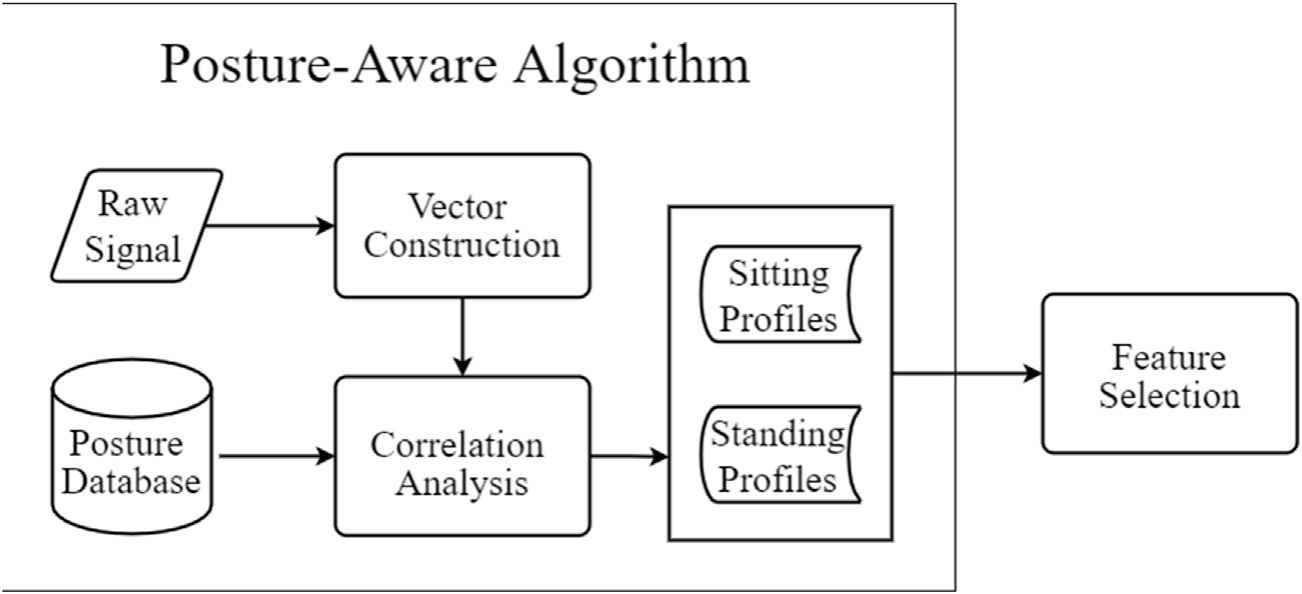
Flowchart of user-independent posture-aware approach.

To tackle this challenge, spider-inspired HCCapture analyzes noise signals to obtain the characteristics of holding postures and identifies victim’s holding posture based on correlation analysis of characteristics. Inspired by voice-print recognition (Liu et al., 2017), we use the characteristics of background noise to detect the holding postures. We first construct noise feature vectors of background noise signals based on commonly used digital signal processing methods, including the power spectral density (PSD) and the mel-frequency cepstral coefficient (MFCC). Then, the correlation analysis is performed on the calculated noise feature vectors and the prior holding posture data. The prior holding posture data contain the noise signals of the two postures we collected in advance. The type of holding posture obtained will be used to complete feature selection. The flowchart of the user-independent posture-aware approach is shown in Figure 4.

### 4.5 Character Restoration

In this phase, we use the handwriting signal segments and posture information to conduct single character recognition. We first extract stroke numbers from handwriting signal segments and obtain a candidate character set in the stroke detection stage. Then, we combine the holding posture information and candidate character set to extract the features in the feature selection stage. Finally, we utilize the speculation model corresponding to the holding posture to determine the character in the *character classification* stage.

In spider-inspired HCCapture, we take the scikit-learn (Scikit-learn, 2007) as the machine learning tool. We train various speculation models for each candidate character set and each holding posture with the neural network algorithm.

1) Stroke detection: Traditional approaches train the speculation model with the collected sensor signals, and use the speculation model to determine the character from the entire alphabet (i.e., the set of 26 characters). However, these approaches suffer from the chirography difference issue as explained in Section 1.

To solve this issue, we employ a generality pattern of characters in handwriting signals, the number of strokes (i.e., the number of peaks in a handwriting signal segment). More specifically, we divide the uppercase alphabet into three clusters according to the number of strokes. The three clusters are

$$\begin{array}{ll} S1 & =\left\{\text{c, l, n, o, s, u, v, C, G, J, L, O, Q, S, U}\right\};\\ S2 & =\left\{\text{a, b, d, e, f, g, h, i, j, p, q, r, s, t, v, w, x, y, z,}\right.\\ & \left.\text{A, B, D, G, J, K, M, N, P, Q, R, S, T, V, W, X, Z}\right\};\\ S3 & =\left\{\text{a, g, h, k, m, n, p, z, A, E, F, H, I, K, N, Y, Z}\right\}. \end{array}.$$

Note that some characters are included in multiple clusters. For example, character “A” is included in both *S*2 and *S*3. This is because the number of character strokes may be uncertain according to different writing habits. However, this situation has limited impact on the subsequent character recognition, since stroke detection aims to generate a candidate character set, which is mainly used to narrow the scope of character speculation.

**FIGURE 5:**
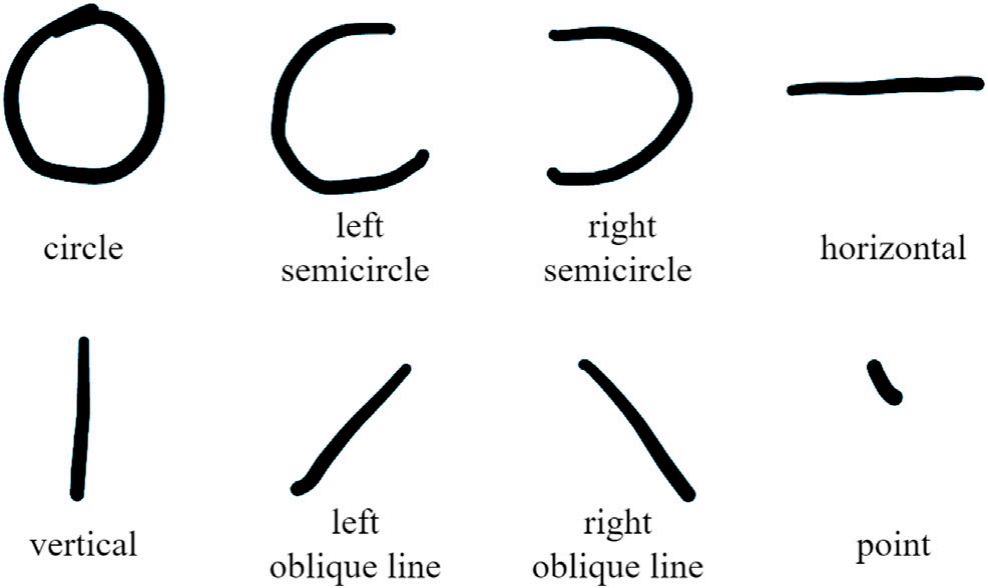
Eight types of strokes that make up a character.

2) Stroke feature: From the signal after noise reduction, the signal of each stroke is extracted with each peak or valley as the boundary. We divide the strokes into: circle, left semicircle, right semicircle, horizontal, vertical, left oblique line, right oblique line, and point, a total of eight types, as shown in Figure 5. Each character is composed of basic strokes, such as “P” can be split into one *vertical* and one *right semicircle.* Then select the appropriate feature subset for training and recognition according to Table 2.
3) Feature selection: When we get the user holding posture and the candidate character set, we first extract the original feature set from the time and frequency domains for a given handwriting signal segment. Then, we execute feature engineering on the segment feature vector, and generate a posture profile. The posture profile is the selected segment feature subset which makes the model achieve the optimal speculation accuracy. In spider-inspired HCCapture, we adopt the *feature selection* module of *scikit-learn* to filter redundant features from the original feature set.

**TABLE 2:** Type of feature extracted from the signal. “*✓*” means that all the features are used, “*ϕ*” means none is used, and “−” means partially used.

Type	Feature	Introduction	Sit	Stand
TD	Std, max, min, mean, and median	Calculate the four characteristics of the three coordinate axes separately	*✓*	−
	Range	Difference between maximum and minimum	*✓*	*ϕ*
	Strength	Expressed by the sum of the squares of the instantaneous readings of the three axes	*✓*	*✓*
FD	Centroid	Indicates where the spectrum centroid is located	*ϕ*	*✓*
	Variance	Display the frequency density of the spectrum	*ϕ*	*✓*
	Skewness	Measuring the asymmetry of the spectrum	*ϕ*	*✓*
	Kurtosis	Describe the size of the range of changes in the spectral values	*✓*	*✓*
	Wiener entropy	Reflecting the flatness of the spectrum of a digital signal	*ϕ*	*✓*

The type of original features extracted from the handwriting signal segment can be seen from Table 2. The original features are calculated from time domain (TD) and frequency domain (FD). It is worth noting that the feature set extracted at this time is the original feature set, and then spider-inspired HCCapture will filter the original feature set according to the user’s holding posture to obtain the most appropriate set of features for each posture.

4) Character classification: Finally, we score the characters in the candidate set and select the most possible character according to the score of each character. Although we use the neural network algorithm to complete the final character speculation at this stage, spider-inspired HCCapture can greatly reduce the impact of the victim’s personal handwriting habits on the accuracy of the speculation model because of the number of strokes.

In addition, the correlation between the written adjacent characters is also taken into account. In this regard, we introduced a Markov model and used the existing corpus (Norvig et al., 2009) for training.

## 5 Experiment Evaluation

In this section, we first introduce the experiment setup and the performance metric adopted. Then, we evaluate the performance of our prototype system spider-inspired HCCapture by executing experiments in a controlled environment. We make performance investigation about handwriting segment detection, and compare it with the detection performance of three other detecting methods. We also conduct a series of experiments to measure the speculation performance changes of spider-inspired HCCapture under different experimental conditions. All the experimental results are presented in this section.

### 5.1 Experiments Setup

We used three devices: Google Pixel 2, Samsung S9, and Xiaomi 10. We recruit 18 volunteers to participate in the experiments. The 18 volunteers (17–32 years old, half for men and women) were equally divided into three groups and used three devices, respectively, marked as Group *A*, *B*, and *C*. Each group is assigned a fixed device.

**FIGURE 6:**
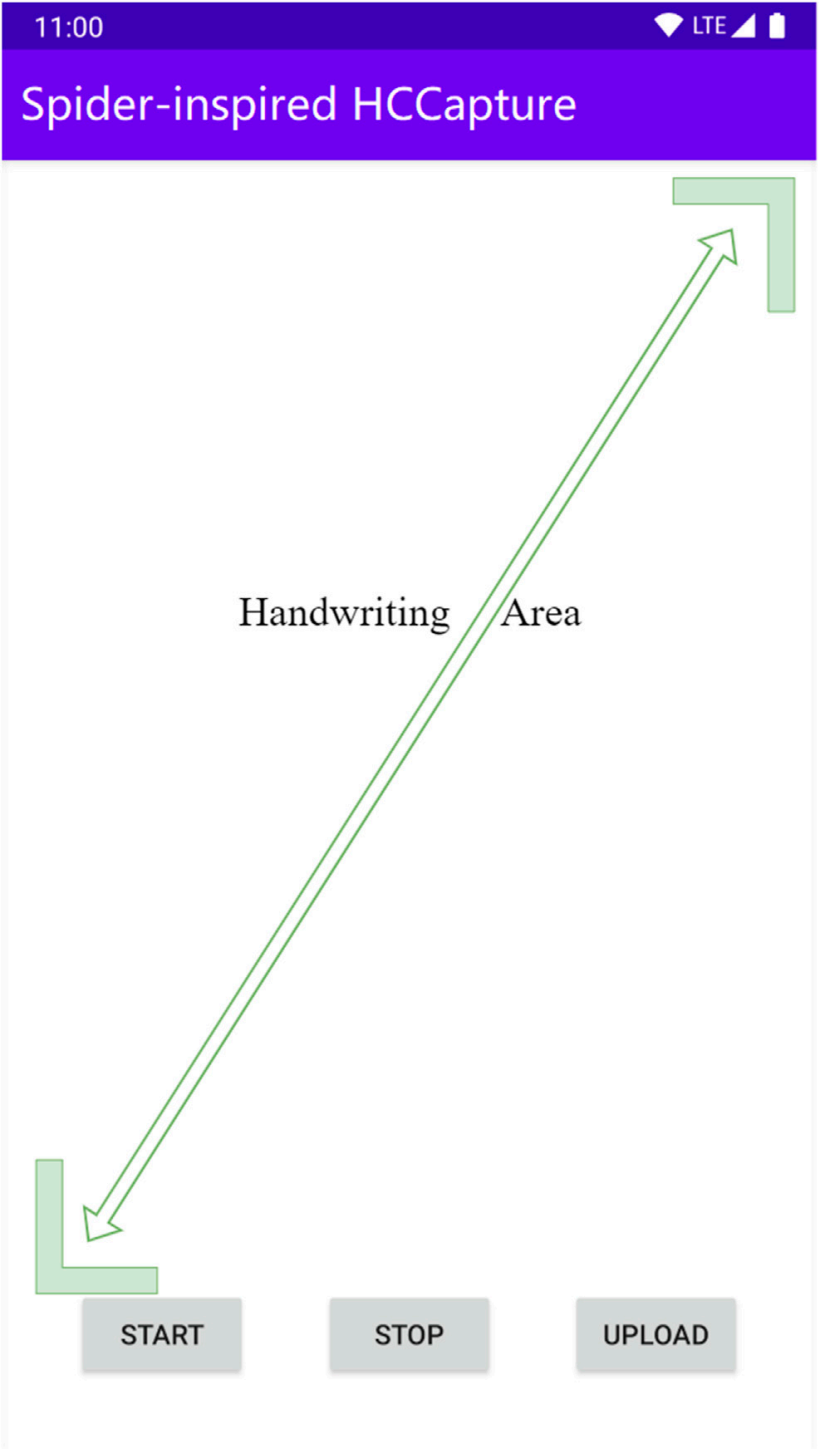
Appearance of Spider-inspired HCCapture App.

Volunteers first boot up our app (Figure 6) and click the *START* button to start signal collection. When characters are handwritten, volunteers click the *STOP* button to stop signal collection. The *UPLOAD* button can send the collected data to the remote server. Each volunteer was asked to write the characters of [*a–zA–Z*] normally 14 times (standing and sitting 7 times each) with their fingers on their own specified device.

In the second round of experiments, volunteers were asked to randomly select 15 words from *Rip Van Winkle* (Irving, 1884) to write, also 14 times (standing and sitting 7 times each).

We use 3-fold cross-validation to verify the accuracy of character inference. Two groups are used as training data, and the third group is used as test data. So, the data from the target user are excluded in the training.

### 5.2 Performance Metric

In our experiment, we use two performance metrics to evaluate the speculation capability of spider-inspired HCCapture: the speculation accuracy and the area under the receiver operating characteristic curve (AUC). Specifically, we define the speculation accuracy as the ratio between the number of correctly speculated characters and the total number of handwritten characters in a testing data. The receiver operating characteristic (ROC) curve describes the false positive rate (FPR) and true positive rate (TPR) under the varying threshold. A higher AUC means that for a given testing data set, spider-inspired HCCapture is able to speculate characters more accurately.

### 5.3 Performance of Segment Detection

**FIGURE 7:**
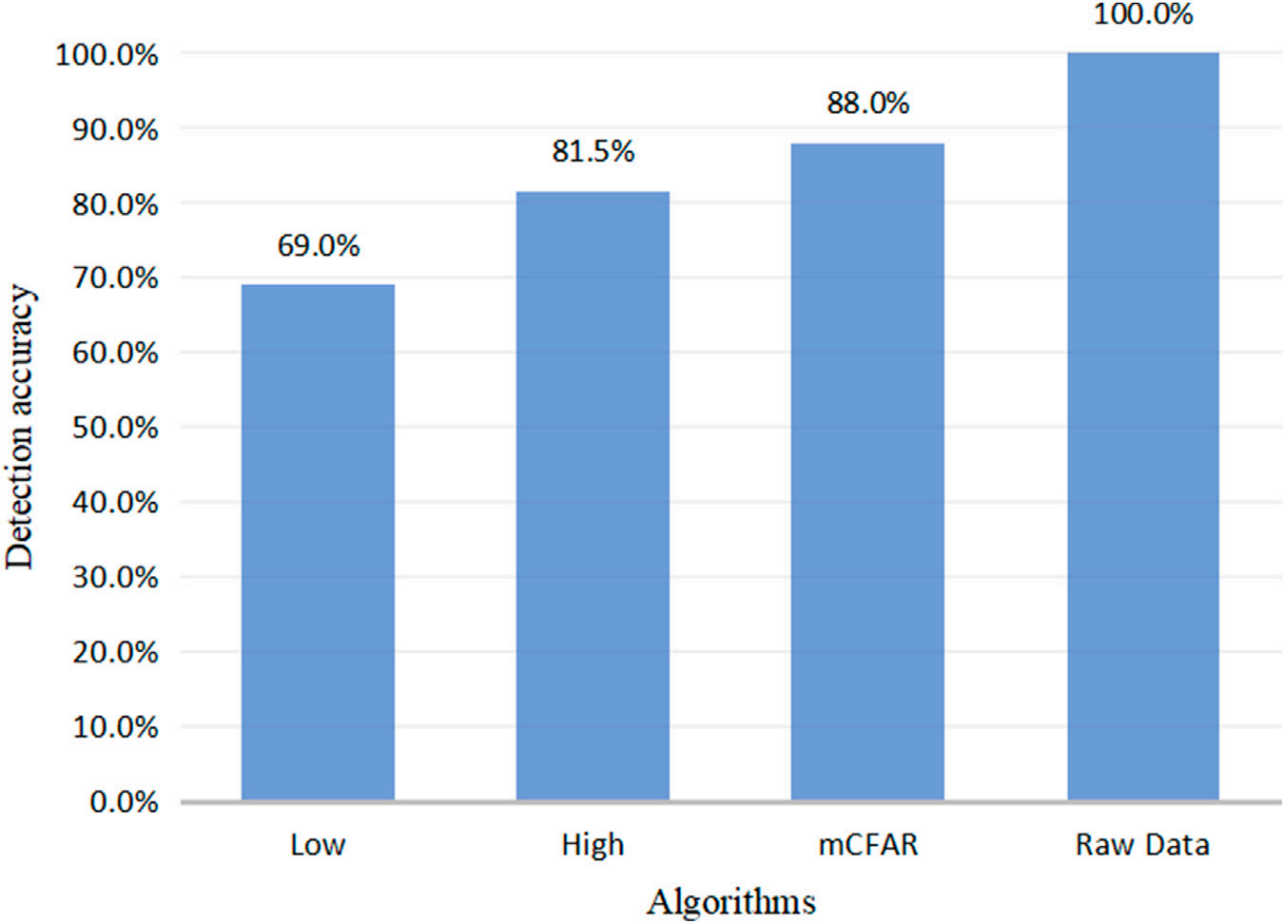
Performance of three segment detection methods.

Figure 7 shows the actual detection result of three segment detection methods. The accuracy here refers to the percentage of the signal data detected by the algorithm to the raw data.

It is obvious that the handwriting segments detected by low threshold are the least, while the detection ability of mCFAR is comparable to that of a high threshold. As sometimes the handwriting strength of volunteers is very weak and cannot produce enough fluctuation changes, mCFAR does not detect all fragments. Considering the whole, we can find that the detection rate of all characters has reached 88%, and it needs to be clear that the character data for subsequent recognition accuracy experiments are based on the character signal that has been detected by the mCFAR.

### 5.4 Performance of Different Holding Postures

**FIGURE 8:**
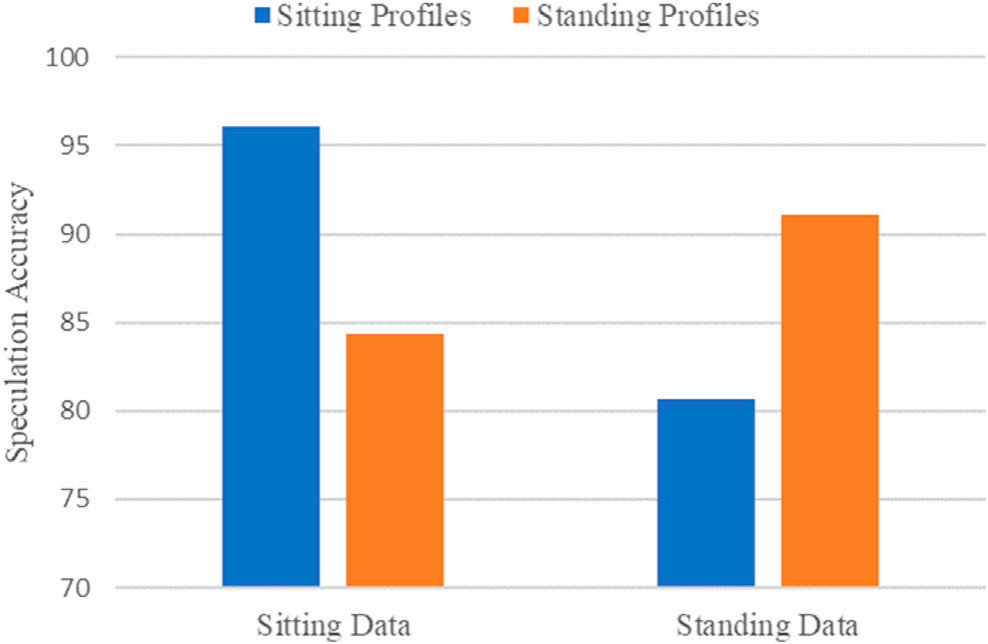
Performance of different holding postures.

Figure 8 shows the speculation accuracy under two different holding postures. We apply each posture profile, generated from the posture-aware analysis, to build the corresponding speculation model and test the speculation accuracy of each model. As lowercase characters were added to the experiment, the speculation space has doubled, so we are allowed to guess twice.

From Figure 8, we can observe that sitting data have the higher speculation accuracy, which can reach 96.1%, and the speculation accuracy of standing data is 91.1%, which are both better than the experimental results (93.5% for sitting, 81.4% for standing) of J. Chen et al. (Chen et al., 2021a). The experimental performance in the sitting posture is better than that in the standing posture, because the sitting posture has less background noise, and we can extract more handwriting segments from the collected sensors’ signals to fully train the speculation model. On the other hand, we can find that when using a posture profile that does not fit the current holding posture to speculate characters, the accuracy is significantly reduced. This is because the posture profile of one holding posture may be redundant and inappropriate for other holding postures, which may reduce the speculation accuracy. The experimental results in the sitting posture will be shown in the follow-up.

### 5.5 Tri-Fold Cross-Validation

**FIGURE 9:**
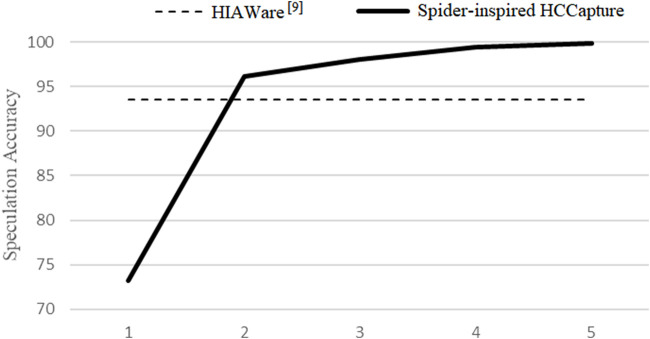
Accuracy: the probability of hitting the correct result, while guesses *n* times in the sitting posture.

First, we evaluated the TPR of guessing *n* times in the 3-fold cross-validation, and the result is shown in Figure 9. Although our experimental speculation space is twice that of J. Chen et al. (Chen et al., 2021a), our TPR is better than theirs within two guesses. The performance degradation of one guess is only 73.2%, maybe caused by too similar capitalization of some characters, such as (*O*, *o*), (*S*, *s*), and (*Z*, *z*). Almost all characters can be speculated within *5 times*, where the accuracy can reach 99.8%.

**FIGURE 10:**
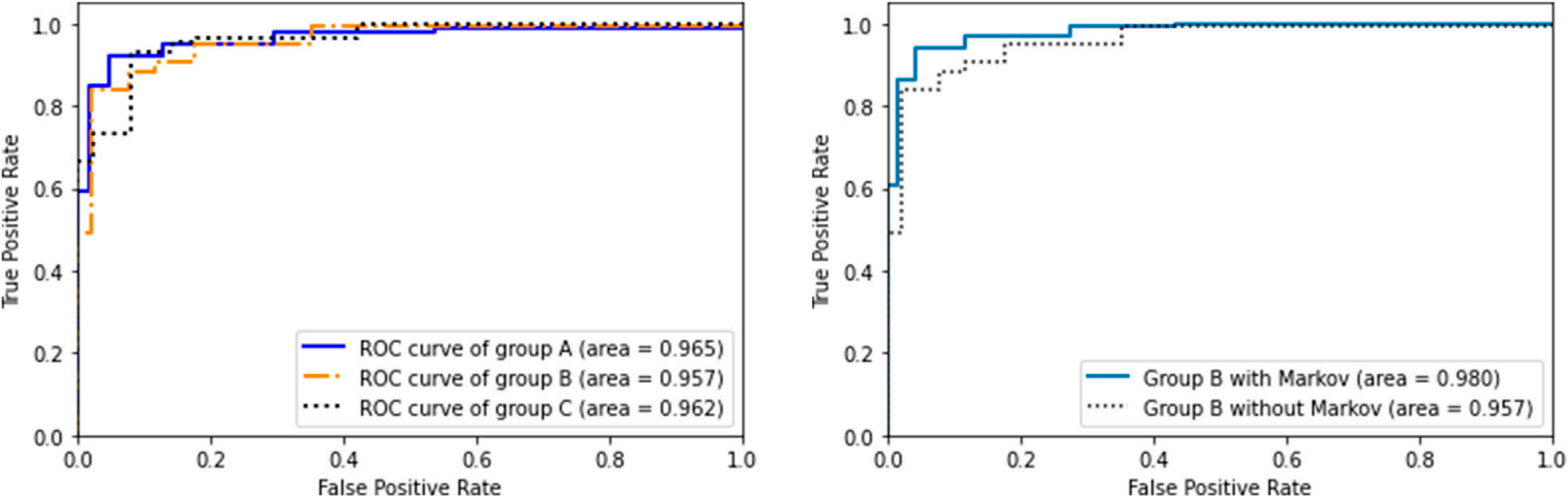
ROC curves of different experiment conditions on spider-inspired HCCapture.

We took two guesses as a representative and drew the ROC curve as shown in Figure 10A. As shown in the figure, the accuracies of the three groups are very close, indicating that spider-inspired HCCapture can smoothly transfer the model to unknown user and device’s data.

### 5.6 Performance of Word

Although the second round of experiments is based on words as the unit of guessing, in order to make the data more intuitive, we calculated the accuracy of each character: $Acc_{c}=\sqrt[{Len_{w}}]{Acc_{w}}$ .

The comparison with the experimental results that do not use the Markov model is shown in the Figure 10B. When there is a correlation between the adjacent characters written, the accuracy of group *B* within *two* guesses increases from 95.7 to 98.0%, which shows that the Markov model can effectively improve the accuracy.

## 6 Conclusion

In this study, we are inspired by the way spiders perceive prey through the subtle vibrations of their webs, and we designed a framework for unrestricted-area handwriting information speculation that is called spider-inspired handwriting character capture (spider-inspired HCCapture), based on the motion sensor signal analysis. We designed a modified constant false alarm rate algorithm (mCFAR) to extract handwriting segments from the motion sensor signal stream, and a user-independent posture-aware approach which combines digital signal processing and correlation analysis. In order to solve the chirography difference issue, spider-inspired HCCapture utilizes a generality pattern of characters (number of strokes and type of strokes). Furthermore, we classify the strokes to obtain a more fine-grained model, which is trained by the neural network algorithm. We also propose a posture-aware approach to solve the posture variation issue. After the user’s posture is recognized, a model specialized for a certain posture is automatically selected to improve the accuracy of the speculation. Moreover, the Markov model is used to deal with the situation where adjacent characters may be correlated when the user writes.

In the experiment, we strictly excluded the target data from the training set and verified it with a 3-fold crossover experiment. When the victim and his device are both unknown, spider-inspired HCCapture can effectively speculate the handwritten contents, with an accuracy rate of 96.1% within *two* guesses. If the number of attempts increases to 5, almost every test data can be successfully predicted.

## Data Availability

The original contributions presented in the study are included in the article/Supplementary Materials, further inquiries can be directed to the corresponding author.
